# Common causes and characteristics of adverse drug reactions in older adults: a retrospective study

**DOI:** 10.1186/s40360-020-00464-9

**Published:** 2020-12-10

**Authors:** Seong-Dae Woo, Jiwon Yoon, Go-Eun Doo, Youjin Park, Youngsoo Lee, So-Hee Lee, Young-Hee Lee, Young-Min Ye

**Affiliations:** 1 Department of Allergy and Clinical Immunology, Ajou University School of Medicine, 206 Worldcup-ro, Yeongtong-gu, Suwon, 16499 Korea; 2 Ajou Regional Pharmacovigilance Center, Ajou University Hospital, Suwon, Korea; 3 Department of Biomedical Sciences, Ajou University Graduate School of Medicine, Suwon, Korea

**Keywords:** Adverse drug reaction, Aged, Drug hypersensitivity, Pharmacovigilance

## Abstract

**Background:**

Aging populations are often accompanied by comorbidity and polypharmacy, leading to increases in adverse drug reactions (ADRs). We sought to evaluate the causes and characteristics of ADRs in older Korean adults (≥65 years) in comparison to younger individuals (< 65 years).

**Methods:**

Of 37,523 cases reported at a Korean pharmacovigilance center from 2011 to 2018, we reviewed 18,842 ADRs of certain or probable causality on the basis of WHO-UMC criteria. We estimated the number of ADRs per 1000 patients exposed to the major culprit drugs, and incidence rate ratios were obtained to assess high- and low-risk medications in older adults.

**Results:**

In total, 4152 (22.0%) ADRs were reported for 3437 older adults (mean age, 74.6 years and 57.3% female). Tramadol (rate ratio, 1.32; 95% confidence interval [CI], 1.21–1.44; *P* < 0.001) and fentanyl (1.49, 1.16–1.92, *P* = 0.002) posed higher risks of ADRs in the older adults, whereas nonsteroidal anti-inflammatory drugs (NSAIDs) (0.35, 0.30–0.40, *P* < 0.001) and iodinated contrast media (ICM) (0.82, 0.76–0.89, *P* < 0.001) posed lower risks. Ratios of serious ADRs to NSAIDs (odds ratio, 2.16; 95% CI, 1.48–3.15; *P* < 0.001) and ICM (2.09, 1.36–3.21, *P =* 0.001) were higher in the older adults than in the younger patients. Analgesics primarily elicited cutaneous ADRs in the younger patients and gastrointestinal reactions in the older adults. ICM more commonly led to anaphylaxis in the older adults than the younger patients (3.0% vs. 1.6%, *P* = 0.019).

**Conclusion:**

For early detection of ADRs in older adults, better understanding of differences in the causes and characteristics thereof in comparison to the general population is needed.

## Background

Pharmacotherapy plays an essential role in the management of older adult patients, but is often accompanied by unexpected adverse drug reactions (ADRs) [1]. Investigators have estimated that the prevalence of ADRs in older adults is approximately 11.0% [2], with ADRs leading to urgent hospitalization in 3.3% [3]. Eliciting considerable morbidity and mortality, ADRs in older adults pose a substantial burden on healthcare costs [2].

Multiple comorbidities in older adults leads to the use of multiple drugs, a condition known as polypharmacy, which increases the risk for adverse drug-drug interactions [4]. Older patients are particularly vulnerable to ADRs because of age-related changes in pharmacokinetics and pharmacodynamics, such as reduced hepatic and renal function, prolonged elimination half-life, and increased sensitivity to drugs [5, 6], which have been shown to be associated with an increased risk of ADRs. However, prescribing drugs to frail older patients can be difficult because of limited evidence on the benefits and risks of medications in the group: medical guidelines on medications are usually based on meta-analyses or randomized clinical trials, which can be biased by the exclusion of older adults, particularly those with comorbidity and polypharmacy [4]. Accordingly, clinicians should prescribe medications with clear therapeutic goals and consider de-prescribing ineffective medications that pose more risk than benefit to minimize inappropriate medication in older patients susceptible to ADRs [7, 8].

In spite of the potential risk of ADRs in older people, only a few studies have explored the epidemiology of ADRs in this population [2, 3, 9]. Moreover, although ADRs should be assessed as part of differential diagnosis in older patients, the consumption of multiple medications accompanied by nonspecific symptoms can make it difficult to identify ADRs and their causes, for which detailed characteristics and drug-specific data are needed. Therefore, this study aimed to evaluate the causes and characteristics of ADRs in patients 65 years of age or older in comparison to younger patients.

## Methods

### Spontaneous reporting ADR database

With efforts to raise awareness of ADR reporting systems and their importance to drug safety surveillance, the Korea Food and Drug Administration (KFDA) has made it mandatory for physicians and pharmacists to report ADRs. Any information on drug safety events can be reported by physicians, pharmacists, nurses, or technicians using standard forms based on electronic medical records (EMRs), and these spontaneous reports are reviewed and evaluated by the pharmacovigilance center employing special trained pharmacists, physicians, and allergy specialists. Information on the reported ADRs is stored in the pharmacovigilance database, and physicians are authorized by pharmacovigilance center can access and utilize the data. Spontaneous reports of ADRs have been collected since the launch of a regional pharmacovigilance center at a tertiary university hospital in 2006. This database includes information on demographic characteristics, medical history, laboratory results, suspected drugs, types and severity of clinical manifestations, dosage, frequency, treatment, causality assessment, and outcomes of ADRs, including the seriousness thereof.

In this study, suspected drugs were grouped according to the first three letters of their Anatomical Therapeutic Chemical (ATC) codes and their chemical substances [10]. Clinical manifestations of ADRs were sorted according to system organ classes (SOC) of the World Health Organization Adverse Reactions Terminology (WHO-ART) [11]. In cases of two or more medications implicated in one adverse event, each medication was evaluated as different ADRs. Each case was evaluated for causality and severity by an evaluation team consisting of special trained nurses, pharmacists, and physicians. Causality was assessed following the World Health Organization-Uppsala Monitoring Center (WHO-UMC) criteria as certain, probable, possible, or unlikely [12]. Severity was described across five categories in accordance with Common Terminology Criteria for Adverse Events (CTCAE) from grade 1 to grade 5, with severity increasing with grade [13]. We identified serious ADRs as events of grades 3–5 based on the CTCAE, including death, life-threatening events, hospitalization (initial or prolonged) due to ADRs, and medically significant ADRs. Underlying diseases were categorized using the International Classification of Disease 10th revision (ICD-10) [14].

### Selection of the ADR database

**Fig. 1:**
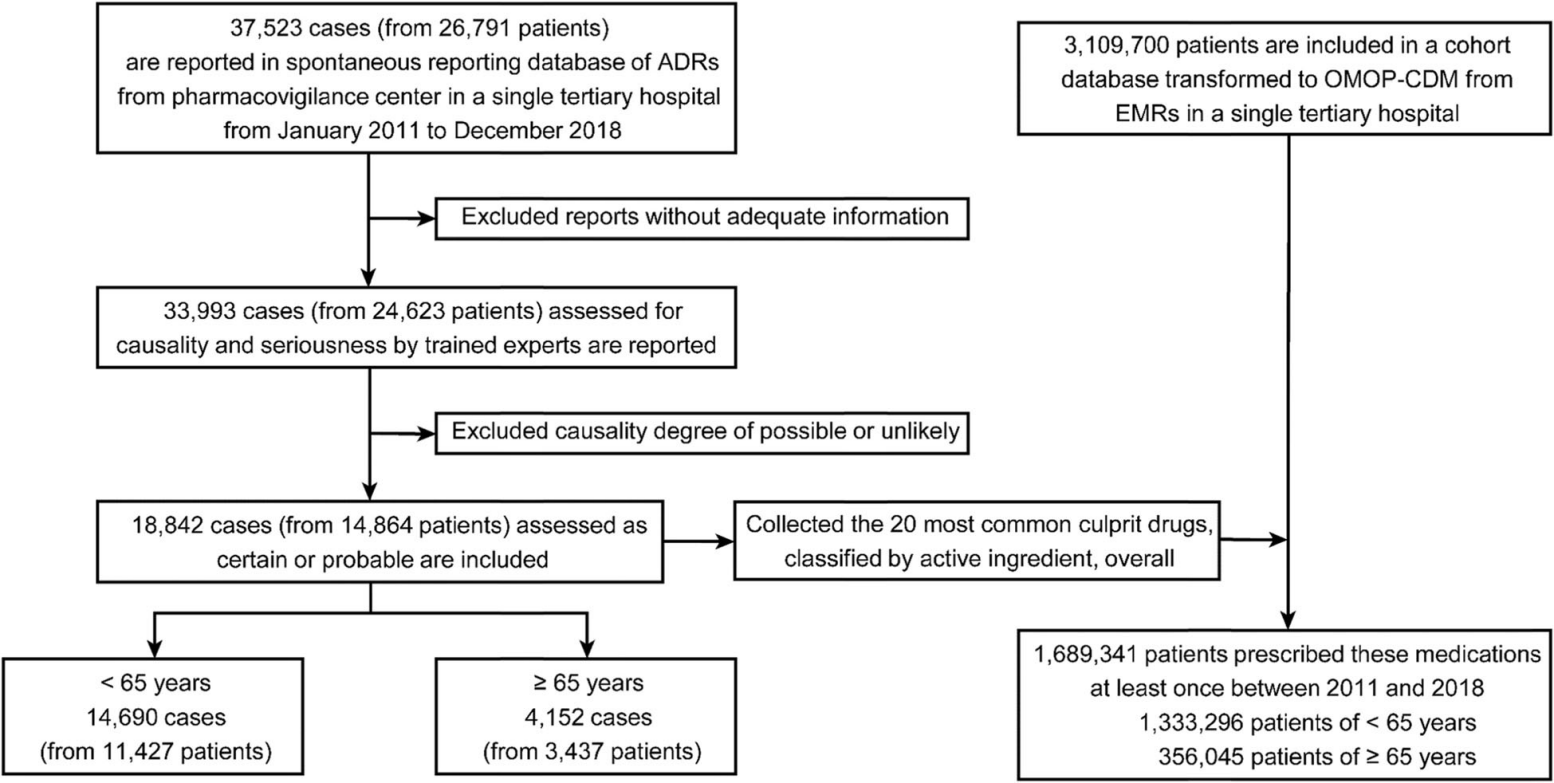
Consort flow of case selection from the database for 2011 to 2018. Causality assessment was performed using the World Health Organization-Uppsala Monitoring Center criteria: certain, probable, possible, or unlikely. ADR, adverse drug reactions; OMOP-CDM, Observational Medical Outcomes Partnership Common Data Model; EMR, electronic medical record

Within each database source, we conducted a retrospective study to investigate ADRs in patients aged ≥65 years at a tertiary care university hospital in Korea. A database containing spontaneously reported ADRs from a pharmacovigilance center were used to gather data for January 2011 to December 2018. A total of 37,523 ADR cases from 26,971 patients were reviewed in the pharmacovigilance database (Fig. 1). Of these, 3530 cases were excluded due to a lack of information for ADR assessment. To raise the relevance and validity of the relationships between suspected drugs and adverse events, we only included 18,842 cases assigned a degree of causality of certain or probable for the current analysis. Also, we classified cases into two groups according to age at the reporting of ADRs: those over 65 years old were defined as the older adult group (4152 cases), and the remaining were defined to the younger patient group (14,690 cases). Overall, 1,689,341 patients were prescribed the 20 most common culprit drugs classified by active ingredient at least once from January 2011 to December 2018.

### The Observational Medical Outcomes Partnership Common Data Model database

Observational Health Data Sciences and Informatics (OHDSI) is an international collaborative that provides a common data model (CDM) for standardizing data from various healthcare databases in regards to terminology and overall structure. The Observational Medical Outcomes Partnership (OMOP) CDM, which maps coding systems into standard terminologies, was developed and is maintained by the OHDSI. In this study, all data from EMRs at a single tertiary hospital were converted into OMOP-CDM format using standard vocabulary concepts to establish a large database transformed to OMOP-CDM including details on patient characteristics, diagnoses, procedures performed, and drugs prescribed.

We ranked the 20 most common drugs classified according to their active ingredients from 18,842 cases assigned a degree of causality of certain or probable in the pharmacovigilance database. As increased medication utilization can result in more reported ADRs, we estimated the number of patients with at least one prescription of the 20 most common culprit drugs from January 2011 to December 2018. The number of ADRs divided by the number of patients prescribed these drugs at least once was calculated to estimate the number of ADR reports per 1000 patients exposed to the major culprit drugs. We obtained incidence rate ratios according to the major culprit drugs to assess high-risk and low-risk medications. We used age, observation period, and prescription medications organized into categories of ingredients for analysis in the OMOP-CDM version 5.3.1. These analyses can provide repeatable and reproducible results.

### Statistical analysis

All statistical analyses were conducted with IBM SPSS, version 25 for Windows (IBM SPSS Inc., Chicago, IL, USA) and R 3.5.2 software (R development core team, http://www.r-project.org). Descriptive statistics are described as frequencies (percentages) for categorical variables and means ± standard deviations for continuous variables. Chi-squared tests with Yates’ correction or the Fisher’s exact test in cases of cells with less than five were used to examine differences between the older and younger patients. Student’s t-test was applied to determine differences in continuous variables between groups. The ratio of serious ADRs to total ADRs was compared between the two groups as odds ratios (OR) with 95% confidence intervals (CI). Also, we estimated the number of ADR reports per 1000 patients exposed to the major culprit drugs, and rate ratios were obtained as the rates in the older adults divided by the rates in the younger individuals to evaluate high-risk and low-risk medications. We calculated rate ratios and 95% CIs according to the categories of culprit drugs, and each estimated result was depicted in a forest plot.

## Results

### Demographics and characteristics of the study population

A total of 18,842 ADR cases in 14,864 patients were included in this study. Of these, 4152 cases (22.0%) were categorized to the older adult group, with the remaining 14,690 cases constituting the younger patient group. The mean age of the older adults was 74.6 years, and 57.3% of them were female (Table 1). The proportions of female patients were similar between the older and younger patients (57.3% vs. 56.4%, *P* = 0.399).

**Table 1:** Baseline demographics and characteristics of the study population

	Total (*N* = 14,864)	< 65 years (*N* = 11,427)	≥ 65 years (*N* = 3437)	*P* value
	18,842	14,690 (78.0)	4152 (22.0)	
Age	49.5 ± 19.2	42.4 ± 15.2	74.6 ± 7.2	< 0.001*
Female	10,897 (57.8)	8496 (57.8)	2401 (57.8)	0.993†
Causality				
Certain	2879 (15.3)	2533 (17.2)	346 (8.3)	< 0.001†
Probable	15,963 (84.7)	12,157 (82.8)	3806 (91.7)	< 0.001†
Severity				
Grade 1	1243 (6.6)	957 (6.5)	286 (6.9)	0.392†
Grade 2	16,606 (88.1)	13,013 (88.6)	3593 (86.5)	< 0.001†
Grade 3	981 (5.2)	714 (4.9)	267 (6.4)	< 0.001†
Grade 4	9 (0.0)	6 (0.0)	3 (0.1)	0.423‡
Grade 5	3 (0.0)	0 (0.0)	3 (0.1)	0.011‡

Values represent numbers of cases with percentage in parentheses. Plus-minus values are means ± standard deviations. *P* values were calculated using *Student’s t-test, † the chi-square test, and ‡ Fisher’s exact test

Based on WHO-UMC causality assessment, 2879 (15.3%) cases of certain and 15,963 (84.7%) of probable causality were identified. There were significant differences in the proportions of certain (8.3% vs. 17.2%, *P* < 0.001) and probable (91.7% vs. 82.8%, *P* < 0.001) ADRs between the older and younger patient groups. The severity of ADRs based on CTCAE was grade 3 (severe or medically significant; hospitalization or prolongation of hospitalization indicated) in 267 older adult cases, which accounted for a higher proportion than that in the younger patients (6.4% vs. 4.9%, *P* < 0.001). The numbers of cases of grade 4 severity (life-threatening or urgent intervention indicated) were three (0.07%) in the older adults and six (0.04%) in the younger patients, with no statistically significant difference (*P* = 0.423); however, all three cases (0.07%) of grade 5 (death related to adverse events) were observed in the older adult group. The most common co-morbid condition among the older adults was neoplasm (26.6%), while that among the younger patients was disease of external causes, such as injury and poisoning (28.4%) (Table 2).

**Table 2:** Comparison of comorbidities in patients with adverse drug reactions between younger patients and older adults

	Total *N* = 14,864 (%)	< 65 years *N* = 11,427 (%)	≥ 65 years *N* = 3437 (%)	*P* value
Comorbid conditions				
Injury, poisoning, and other consequences of external causes	3771 (20.0)	3246 (28.4)	525 (15.3)	< 0.001
Diseases of the digestive system	3496 (18.6)	2676 (23.4)	820 (23.9)	0.594
Neoplasms	3375 (17.9)	2462 (21.5)	913 (26.6)	< 0.001
Diseases of the respiratory system	2744 (14.6)	2207 (19.3)	537 (15.6)	< 0.001
Diseases of the skin and subcutaneous tissue	2112 (11.2)	1807 (15.8)	305 (8.9)	< 0.001
Diseases of the circulatory system	2057 (10.9)	1222 (10.7)	835 (24.3)	< 0.001
Diseases of the musculoskeletal system and connective tissue	1821 (9.7)	1313 (11.5)	508 (14.8)	< 0.001
Diseases of the genitourinary system	1666 (8.8)	1244 (10.9)	422 (12.3)	0.028
Certain infectious and parasite disease	1644 (8.7)	1304 (11.4)	340 (9.9)	0.010
Endocrine, nutritional, and metabolic diseases	1558 (8.3)	1087 (9.5)	471 (13.7)	< 0.001
Diseases of the nervous system	1228 (6.5)	959 (8.4)	269 (7.8)	0.283

Values represent numbers of patients with percentages in parentheses. Plus-minus values are means ± standard deviations. *P* values were obtained from the chi-square test with Yates’ correction

### Culprit agents

**Fig. 2:**
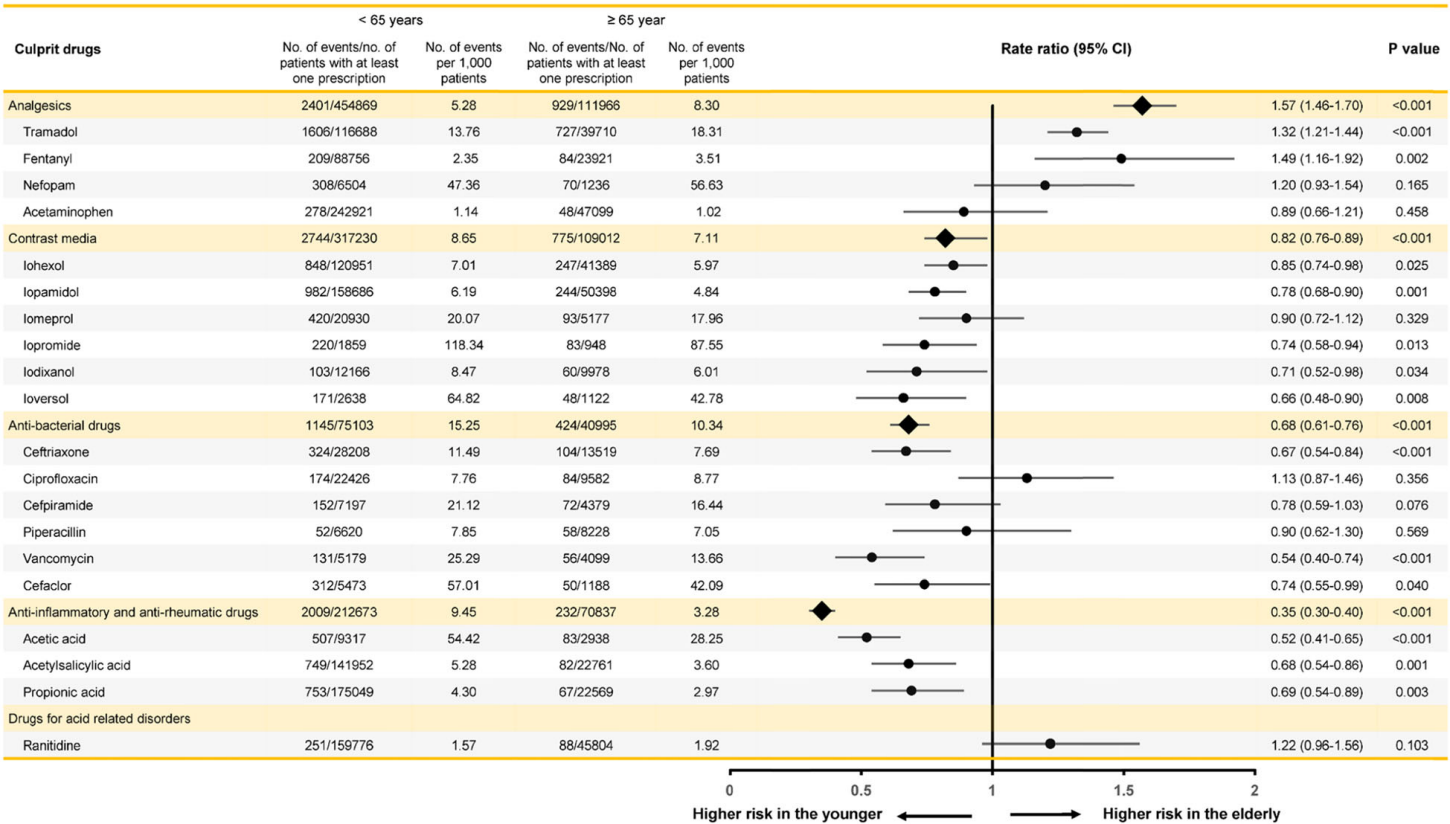
Rate ratios of ADR reports per 1000 patients exposed to the 20 most frequently culprit drugs classified by active ingredient. Forest plot depicts relative risk and 95% confidence intervals according to culprit drugs. CI, confidence interval

The categories of the most commonly implicated culprit agents were analgesic drugs (22.4%), contrast media (20.1%), anti-bacterial drugs (19.5%), anti-inflammatory and anti-rheumatic drugs (8.7%), and drugs for acid-related disorders (3.8%) (Table S1 in the Supplementary Appendix). We ranked the 20 most common drugs classified by active ingredient and estimated the number of ADR reports per 1000 patients exposed to these drugs in the older and younger individuals (Fig. 2). ADRs induced by these common culprit drugs accounted for nearly three-fifths of all reported cases (10,998 of 18,842, 58.4%). In regards to rate ratio (RR) compared to the younger patients, tramadol (RR 1.57, 95% CI 1.46–1.70, *P* < 0.001) and fentanyl (RR 1.32, 95% CI 1.21–1.44, *P* < 0.001) were the top-ranked culprits for ADRs in the older adults. ICM, including iohexol, iopamidol, iomeprol, iopromide, iodixanol, and ioversol, posed a lower RR in the older adults, compared with the younger patients (RR 0.82, 95% CI 0.76–0.89, *P* < 0.001). The RR for NSAIDs, including acetic acid, acetylsalicylic acid, and propionic acid, was significantly lower in the older adults than in the younger patients (RR 0.35, 95% CI 0.30–0.40, *P* < 0.001). Of commonly implicated antibiotics, ceftriaxone (RR 0.67, 95% CI 0.54–0.84, *P* < 0.001), vancomycin (RR 0.54, 95% CI 0.40–0.74, *P* < 0.001), and cefaclor (RR 0.74, 95% CI 0.55–0.99, *P* = 0.040) exhibited a lower risk in the older adults than in the younger individuals.

The ratios of serious ADRs (CTCAE grade 3–5) to total ADRs for the 20 most common culprit drugs are shown in Table 3. We found that ICM (OR 2.09, 95% CI 1.36–3.21, *P =* 0.001) and NSAIDs (OR 2.16, 95% CI 1.48–3.15, *P* < 0.001) elicited significantly higher rates of serious ADRs in the older adults than in the younger patients. Among antibiotics, ceftriaxone showed a relatively higher rate of serious ADRs in the older adults (OR 2.75, 95% CI 1.28–5.93, *P =* 0.008) than in the younger patients, whereas cefaclor showed a significantly lower rate of serious ADRs (OR 0.04, 95% CI 0.01–0.13, *P* < 0.001).

**Table 3:** Ratios of serious ADRs to total ADRs for the 20 most common culprit drugs classified by active ingredient

Culprit drugs	< 65 years	≥ 65 years	Odds ratio (95% CI)	*P* value
	Serious / Total ADRs (%)			
Analgesics	41/2401 (1.7)	16/929 (1.7)	1.01 (0.56–1.81)	0.977
Tramadol	24/1606 (1.5)	10/727 (1.4)	0.92 (0.44–1.93)	0.824
Fentanyl	3/209 (1.4)	3/84 (3.6)	2.54 (0.50–12.86)	0.243
Nefopam	0/308 (0)	0/70 (0)	N/A	N/A
Acetaminophen	14/278 (5.0)	3/48 (6.3)	1.26 (0.35–4.55)	0.727
Contrast media	59/2744 (2.2)	34/775 (4.4)	2.09 (1.36–3.21)	0.001
Iohexol	4/848 (0.5)	9/247 (3.6)	7.98 (2.44–26.14)	< 0.001
Iopamidol	24/982 (2.4)	7/244 (2.9)	1.18 (0.50–2.77)	0.705
Iomeprol	7/420 (1.7)	6/93 (6.5)	4.07 (1.34–12.40)	0.008
Iopromide	16/220 (7.3)	4/83 (4.8)	0.65 (0.21–1.99)	0.443
Iodixanol	2/103 (1.9)	5/60 (8.3)	4.59 (0.86–24.45)	0.052
Ioversol	6/171 (3.5)	3/48 (6.3)	1.83 (0.44–7.62)	0.398
Anti-bacterial drugs	60/864 (6.9)	37/424 (8.7)	1.28 (0.84–1.96)	0.255
Ceftriaxone	16/324 (4.9)	13/104 (12.5)	2.75 (1.28–5.93)	0.008
Ciprofloxacin	8/174 (4.6)	5/84 (6.0)	1.31 (0.42–4.14)	0.641
Cefpiramide	1/152 (0.7)	1/72 (1.4)	2.13 (0.13–34.49)	0.587
Piperacillin	3/52 (5.8)	7/58 (12.1)	2.24 (0.55–9.17)	0.251
Vancomycin	10/131 (7.6)	7/56 (12.5)	1.73 (0.62–4.08)	0.289
Cefaclor	22/31 (71.0)	4/50 (8.0)	0.04 (0.01–0.13)	< 0.001
Anti-inflammatory and anti-rheumatic drugs	172/2009 (8.6)	39/232 (16.8)	2.16 (1.48–3.15)	< 0.001
Acetic acid	40/507 (7.9)	10/83 (12.0)	1.60 (0.77–3.34)	0.207
Acetylsalicylic acid	90/749 (12.0)	19/82 (23.2)	2.21 (1.26–3.86)	0.005
Propionic acid	42/753 (5.6)	10/67 (14.9)	2.97 (1.42–6.23)	0.003
Drugs for acid-related disorders				
Ranitidine	15/251 (6.0)	5/88 (5.7)	0.95 (0.33–2.69)	0.920

Values represent numbers of cases with percentages in parentheses. *P* values were obtained from the chi-square test

*ADR* Adverse drug reaction, *CI* Confidence interval, *N/A* Not applicable

### Clinical manifestations

The clinical manifestations of ADRs appeared to vary with age. Skin disorders were the most common manifestations of ADRs overall (45.3%), followed by gastrointestinal disorders (26.8%) and nervous system disorders (12.6%) (Table S2 in the Supplementary Appendix). Skin disorders were the most commonly documented ADRs associated with analgesics among the younger patients (53.9%), whereas gastrointestinal disorders were most common among the older adults (68.3%) (Table 4). While the occurrence rates of cutaneous manifestations (25.9% vs. 53.9%, *P* < 0.001) and gastrointestinal disorders (68.3% vs. 47.7%, *P* < 0.001, respectively) in relation to analgesics differed significantly between the older adults and younger groups, no significant differences were observed in common symptoms of ADRs associated with ICM and antibiotics between the two groups. ICM was more strongly associated with heart-related disorders (6.1% vs. 2.6%, *P* < 0.001) and anaphylaxis (3.0% vs. 1.6%, *P* = 0.019) in the older adults than in the younger individuals.

**Table 4:** Clinical manifestations of ADRs according to causative drugs categorized by ATC code

WHO-ART SOC	Analgesic drugs			Contrast media			Antibiotics		
	< 65 *n* = 4083 (%)	≥ 65 *n* = 1161 (%)	*P* value	< 65 *n* = 2737 (%)	≥ 65 *n* = 757 (%)	*P* value	< 65 *n* = 2521 (%)	≥ 65 *n* = 710 (%)	*P* value
Skin	2200 (53.9)	301 (25.9)	< 0.001	2337 (85.4)	628 (83.0)	0.174	1653 (65.6)	427 (60.1)	0.878
Gastrointestinal	1947 (47.7)	793 (68.3)	< 0.001	341 (12.5)	98 (12.9)	0.843	982 (39.0)	252 (35.5)	1.000
Nervous system	781 (19.1)	228 (19.6)	0.067	231 (8.4)	53 (7.0)	0.207	286 (11.3)	75 (10.6)	0.907
General	534 (13.1)	110 (9.5)	0.035	319 (11.7)	86 (11.4)	0.807	404 (16.0)	94 (13.2)	0.409
Respiratory	648 (15.9)	112 (9.6)	< 0.001	291 (10.6)	67 (8.9)	0.156	339 (13.4)	60 (8.5)	0.007
Heart-related	198 (4.8)	68 (5.9)	0.039	70 (2.6)	46 (6.1)	< 0.001	131 (5.2)	27 (3.8)	0.344
Anaphylaxis	240 (5.9)	52 (4.5)	0.302	44 (1.6)	23 (3.0)	0.019	234 (9.3)	41 (5.8)	0.025
SCARs	5 (0.1)	4 (0.3)	0.090	4 (0.1)	1 (0.1)	1.000	14 (0.6)	7 (1.0)	0.170

Values represent numbers of patients with percentages in parentheses. *P* values were obtained from the chi-square test with Yates’ correction

*ADR* Adverse drug reaction, *WHO-ART* World Health Organization Adverse Reactions Terminology, *SOC* System Organ Classes, *ATC* Anatomical Therapeutic Chemical, *SCAR* Severe cutaneous adverse reaction

## Discussion

On the basis of reports from a pharmacovigilance center at a single tertiary hospital in South Korea from 2011 through 2018, we estimated the characteristics and culprit agents of ADRs in patients 65 years of age or older. We found that the causes and clinical features of ADRs in the older adults differed considerably from those in younger patients, with observable differences in the manifestations of adverse reactions depending on the culprit drugs. We found tramadol and fentanyl to be the most frequently reported culprit drugs in the older adults, compared to the younger individuals: the high prevalence of older patients with neoplasms may account for the high number of ADRs related with analgesics, such as tramadol, fentanyl, and nefopam. Even though the frequencies of ADRs caused by NSAIDs and ICM were lower in the older adults than in their younger counterparts, the rates of serious ADRs to NSAIDs and ICM were significantly higher in the older adults than in the younger individuals. ICM was found to be related to higher risks of anaphylaxis and heart-related disorders in the older adults than in the younger individuals.

People aged 65 years and older are now the most rapidly growing population in the world and are particularly susceptible to ADRs because of multiple comorbidities, the use of multiple drugs, and age-related changes in pharmacokinetics and pharmacodynamics. Advancing age is associated with an increased prevalence of multiple morbidities, inevitably leading to the concurrent use of multiple medications. Polypharmacy can lead to increased risks of adverse drug-drug and drug-disease interactions, inappropriate medication use, under-use of effective treatment, poor medication adherence, and most importantly adverse drug events [4]. Despite its dangers, there is evidence of rising rates of polypharmacy with potentially drug-drug interactions in older patients [15]. While polypharmacy is likely inevitable, in many cases, it may be due to inappropriate prescription of medications and preventable problems [16]. Therefore, physicians should regularly review and optimize medications to reduce unnecessary polypharmacy. They can prescribe safer alternatives when available and use lower doses for shorter durations, or can take measures to minimize adverse events with prescribing prophylactic medications and intensifying patient education [17]. Also, they should prescribe new medications with clear therapeutic goals and consider the risk-to-benefit profiles when prescribing them to older patients. In addition, age-related physiological changes in pharmacokinetics and pharmacodynamics have significant clinical implications. With age, water content declines, while fat content rises, which influences the volume of distribution of drugs in older adults [18]. Also, renal drug excretion and hepatic drug metabolism are reduced with aging: these changes can result in prolonged elimination half-life and drug accumulation [7, 18]. These factors make older patients more vulnerable to drug-drug interactions than younger individuals. Finally, older patients tend to be more sensitive to the effects of medications than younger individuals because of altered pharmacodynamics responses, which are generally predictable and can be minimized by titrating carefully from a low starting dose [7].

Korea offers universal access to health care, regardless of one’s ability to pay, through the National Health Insurance and Medical Aid program. All citizens can receive appropriate healthcare services, including examination, surgery, medication, etc. Research has shown that universal health coverage of medical services is associated with increased use of prescription drugs, which can result in an increased number of reported ADRs. We reviewed a large database of ADRs of certain or probable causality documented at a pharmacovigilance center. Similar with prior reports [19, 20], we noted 22.0% of all ADR cases occurred in adults older than 65 years. Notably, the prevalence of ADRs was higher in females than in males in both the older and younger patients. These results are in line with previous studies indicating that older age and female sex are associated with an increased risk for the development of ADRs [20, 21]. A higher prevalence of ADRs in females has been suggested as being related to differences in propensity for symptom reporting, drug prescription rates, medical care utilization, hormonal factors, and pharmacokinetic factors [19]. We also documented a higher rate of severe ADRs of grades 3–5 based on CTCAE in the older adults than in the younger patients. Accordingly, we suspect that more complicated comorbidities and lower tolerance to adverse reactions in older adults can elicit more frequent occurrences of severe ADRs.

Studies on ADRs in older adults over the past few decades have shown divergent results on causative agents and clinical features. Accounting for the heterogeneity between studies may be variations in how researchers have defined and assessed ADRs [21]. Moreover, demographics, prevalent diseases, economic states, genetic differences, and prescription patterns in different countries can influence the characteristics of ADRs. In the present study, the most common culprit drugs for ADRs in the older adults were analgesics, contrast media, antibiotics, and NSAIDs, results that are similar with those at other pharmacovigilance centers in Korea [19, 22, 23]. Cutaneous and gastrointestinal disorders were the most frequent manifestations in our study, with significantly different occurrence rates between the older and younger patients, which is consistent with the results of previous studies in Korea [20, 22, 23]. Meanwhile, results on causative drugs and clinical manifestations differ among various clinical settings and countries. A previous study in Spain showed that the most common ADRs leading to hospitalization were acute renal failure induced by renin-angiotensin system inhibitors, gastrointestinal bleeding related to anti-thrombotics and/or NSAIDs, and intracranial bleeding caused by vitamin K antagonists [3]. Another study in Canada reported that the two most common drug categories implicated in hospitalizations for ADRs in older adults were cardiovascular agents and analgesics/anti-inflammatory drugs [24]. Hospitalization after emergency department visits for adverse drug events in older Americans resulted most commonly from warfarin, insulins, oral antiplatelet agents, and oral hypoglycemic agents [25].

Serious ADRs leading to morbidity, mortality, and high healthcare costs are a major concern. In the present study, the two most common drug categories implicated in serious ADRs in the older adults were NSAIDs and antibiotics, similar to two retrospective studies of a spontaneous reporting database at a pharmacovigilance center [19, 26]. Accordingly, physicians should consider the risk-to-benefit profiles of these drugs when prescribing them [27]. The older adults in this study also experienced nearly twice as many anaphylactic reactions caused by ICM, compared with the younger group. Similarly, a recent study also found that older patients over 60 years were more likely to experience anaphylaxis due to nonionic low osmolality contrast media [28]. With the recent increase in the use of computed tomography, ICM use has also steadily increased. Although ICM is administered once in conjunction with CT scans, whereas most prescribed medications are generally used for several days, our results showed that ICM was a common culprit drug for ADRs. Thus, physicians should be aware of the potential risks posed by ICM to older adults and be prepared to administer appropriate emergency management of the adverse events associated with the use thereof.

Several studies have been performed in regards to the epidemiology, causative drugs, and risk factors associated with ADRs in older adults. However, to our knowledge, few have investigated the characteristics of ADRs in older adults in comparison to those in younger individuals, which is the strength of the current study. Furthermore, previous studies using databases of ADRs have only analyzed numbers of adverse events, the use of which may not be reliable, because the prescription of several drugs at a time can result in an increased number of reported ADRs. We, however, used ADR reports per 1000 patients to assess high- and low-risk medications in older adults to obtain reliable numbers of ADR cases and patients taking at least one medication during the study period. In addition, we only included ADR cases of certain or probable causality based on the WHO-UMC criteria, supporting the relevance and validity of the relationships between the culprit drugs and adverse events.

Despite the strengths above, there are several limitations to the present study. First, this study relied on spontaneously reported ADRs, which may pose some underestimation of ADRs, since adverse events are underreported by clinicians and nurses. Also, as a retrospectively designed study, we were not able to reduce possible bias caused by missing data. Second, there could be potential bias in causality assessment evaluated by physicians or pharmacists. Though these processes are not perfect, we also used diagnostic tools, such as skin testing, blood tests measuring specific immunoglobulin E, and drug provocation tests. Third, our findings on the most commonly reported causative agents and clinical symptoms may be divergent from other populations with different prescribing patterns, disease epidemiology, and ethnicities. Fourth, we did not collect data on adverse events associated with non-prescription drugs, such as complementary or alternative medications, although these drugs are commonly used in older adults. Alternative medications may lead to serious adverse events, including palpitations, chest pain, or hepatotoxicity, as well as potential interactions with prescriptions drugs [6, 29]. Further studies are needed to increase awareness of the potential risks of non-prescription drugs among physicians.

## Conclusion

In conclusion, our findings suggest that the characteristics of ADRs, particularly their causes and clinical manifestations, in older adults are markedly different from those of younger individuals. Since no ideal tool for the assessment of ADR exists, clinical judgement based on a history of drug administration and their reactions is necessary for detecting an ADR. Knowledge of the most frequently responsible culprits and clinical manifestations of ADRs in older adults will be beneficial to the early detection and prevention of them.

## Supplementary Information

**Additional file 1: Table S1.** The most commonly implicated drugs categorized by ATC code. **Table S2.** Clinical manifestations of ADRs classified according to WHO-ART SOC.

## Data Availability

The datasets used and analyzed during the current study are available from the corresponding author upon reasonable request.

## Abbreviations

ADR: Adverse drug reaction
NSAID: Nonsteroidal anti-inflammatory drug
ICM: Iodinated contrast media
KFDA: Korea Food and Drug Administration
EMR: Electronic medical record
ATC: Anatomical Therapeutic Chemical
SOC: System organ class
WHO-ART: World Health Organization Adverse Reactions Terminology
WHO-UMC: World Health Organization-Uppsala Monitoring Center
CTCAE: Common Terminology Criteria for Adverse Event
ICD-10: International Classification of Disease 10th revision
OHDSI: Observational Health Data Sciences and Informatics
CDM: Common data model
OMOP: Observational Medical Outcomes Partnership
