# Supplementary material for: Common causes and characteristics of adverse drug reactions in older adults: a retrospective study
Source: BMC Pharmacol Toxicol. 2020 Dec 10;21:87. doi: 10.1186/s40360-020-00464-9 (PMC7727226; doi:10.1186/s40360-020-00464-9)
Supplement: Supplementary file 1 — Additional file 1: Table S1. The most commonly implicated drugs categorized by ATC code. Table S2. Clinical manifestations of ADRs classified according to WHO-ART SOC. [file 40360_2020_464_MOESM1_ESM.docx]

**Table S1. The most commonly implicated drugs categorized by ATC code**

|  | ATC code | Total  n=14,864 (%) | < 65 years  n=11,427 (%) | ≥ 65 years  n=3,437 (%) |
| --- | --- | --- | --- | --- |
|  |  | 18842 | 14690 | 4152 |
| Analgesic drugs | N02 | 4214 (22.4) | 3159 (21.5) | 1055 (25.4) |
| Contrast media | V08 | 3785 (20.1) | 2960 (20.1) | 825 (19.8) |
| Anti-bacterial drugs | J01 | 3681 (19.5) | 2881 (19.6) | 800 (19.3) |
| Anti-inflammatory and anti-rheumatic drugs | M01 | 1642 (8.7) | 1450 (9.9) | 192 (4.6) |
| Drugs for acid related disorders | A02 | 716 (3.8) | 550 (3.7) | 166 (4.0) |
| Anti-epileptic drugs | N03 | 578 (3.1) | 511 (3.5) | 67 (1.6) |
| Drugs for functional gastrointestinal disorders | A03 | 385 (2.0) | 331 (2.2) | 54 (1.3) |
| Anesthetic drugs | N01 | 253 (1.3) | 190 (1.3) | 63 (1.5) |
| Anti-neoplastic drugs | L01 | 232 (1.2) | 160 (1.1) | 72 (1.7) |
| Anti-thrombotic agents | B01 | 213 (1.1) | 151 (1.0) | 62 (1.5) |
| Cough and cold drugs | R05 | 199 (1.1) | 152 (1.0) | 47 (1.1) |
| Anti-histamines for systemic use | R06 | 183 (1.0) | 148 (1.0) | 35 (0.8) |
| Psycholeptic drugs | N05 | 172 (0.9) | 120 (0.8) | 52 (1.3) |
| Anti-mycobacterial drugs | J04 | 167 (0.9) | 109 (0.7) | 58 (1.4) |
| Calcium channel blockers | C08 | 107 (0.6) | 62 (0.4) | 45 (1.1) |
| Agents acting on the renin-angiotensin system | C09 | 101 (0.5) | 58 (0.3) | 43 (1.0) |
| Psychoanaleptics | N06 | 95 (0.5) | 53 (0.3) | 42 (1.0) |
| Lipid modifying agents | C10 | 86 (0.5) | 53 (0.3) | 33 (0.8) |
| Blood substitutes and perfusion solutions | B05 | 83 (0.4) | 52 (0.3) | 31 (0.7) |
| Diuretic drugs | C03 | 50 (0.3) | 19 (0.1) | 31 (0.7) |

Values represent numbers of cases with percentages in parentheses. ATC, anatomical therapeutic chemical.

**Table S2. Clinical manifestations of ADRs classified according to WHO-ART SOC**

|  | Total  n=14,864 (%) | < 65 years  n=11,427 (%) | ≥ 65 years  n=3,437 (%) | *P* value |
| --- | --- | --- | --- | --- |
| WHO-ART SOC | 18842 | 14690 | 4152 |  |
| Skin and Appendages Disorders | 8530 (45.3) | 6940 (47.2) | 1590 (38.3) | <0.001 |
| Gastro-intestinal system disorders | 5049 (26.8) | 3695 (25.2) | 1354 (32.6) | <0.001 |
| Central and peripheral Nervous System Disorders | 2378 (12.6) | 1817 (12.4) | 561 (13.5) | 0.055 |
| Body as a Whole General Disorders | 1963 (10.4) | 1536 (10.5) | 427 (10.3) | 0.749 |
| Respiratory system disorders | 1730 (9.2) | 1419 (9.7) | 311 (7.5) | <0.001 |
| Heart related disorders | 761 (4.0) | 540 (3.7) | 221 (5.3) | <0.001 |
| Anaphylaxis | 654 (3.5) | 521 (3.5) | 133 (3.2) | 0.273 |
| Psychiatric Disorders | 401 (2.1) | 309 (2.1) | 92 (2.2) | 0.658 |
| Blood related disorders | 298 (1.6) | 204 (1.4) | 94 (2.3) | <0.001 |
| Urinary system disorders | 249 (1.3) | 172 (1.2) | 77 (1.9) | 0.003 |
| Liver and biliary system disorders | 188 (1.0) | 157 (1.1) | 31 (0.7) | 0.042 |
| Musculoskeletal System Disorders | 152 (0.8) | 111 (0.8) | 41 (1.0) | 0.171 |
| Liver and biliary system disorders | 188 (1.0) | 157 (1.1) | 31 (0.7) | 0.042 |
| Vision Disorders | 119 (0.6) | 104 (0.7) | 15 (0.4) | 0.003 |
| Application site disorders | 68 (0.4) | 44 (0.3) | 24 (0.6) | 0.027 |
| Vascular (extracardiac) disorders | 67 (0.4) | 50 (0.3) | 17 (0.4) | 0.509 |
| SCAR | 36 (0.2) | 27 (0.2) | 9 (0.2) | 0.668 |
| Others | 90 (0.5) | 74 (0.5) | 16 (0.4) | 0.329 |

Values represent numbers of cases with percentages in parentheses. *P* values were obtained from the chi-square test with Yates’ correction. ADR, adverse drug reaction; WHO-ART, World Health Organization Adverse Reactions Terminology; SOC, system organ classes.
